# Rolling in the deep: therapeutic targeting of circulating tumor cells

**DOI:** 10.3389/fonc.2012.00184

**Published:** 2012-11-29

**Authors:** Michael R. King

**Affiliations:** Department of Biomedical Engineering, Cornell UniversityIthaca, NY, USA

*Circulating tumor cells (CTCs)*. What exactly are these cells that we liken to “finding a needle in a haystack?” CTCs originate from a primary tumor, are often but not always of epithelial phenotype, and are dispersed in the peripheral circulation among millions of (somewhat smaller) leukocytes per milliliter and billions of red blood cells. They can and do interact with the proteins and formed elements of blood. They are the subject of rapidly increasing interest in research and clinical diagnostics. But are they the same cells that initiate metastases? Recent improvements to animal models of bloodborne metastasis have begun to elucidate the physical determinants of tissue tropism in metastasis. One thing for certain is that CTC count is now recognized as a strong predictor of patient survival in many cancers, such as those originating from breast and prostate. This Special Topic of Frontiers of Oncology attempts to address some of the important questions surrounding CTCs: what are these cells, how may we study and target them therapeutically, and what secrets they may hold in controlling and combating cancer metastasis.

Eleven of the 15 papers in this Special Topic come from investigators of the collaborative network of Physical Science-Oncology Centers established in 2009 by the US National Cancer Institute. These 12 centers across the United States bring together biologists, clinicians, physical scientists, and engineers to develop innovative new ways to understand and fight cancer. The specific centers represented here include: the Cornell University Center on the Microenvironment and Metastasis; the Center for Transport Oncophysics of the Methodist Hospital Research Institute; the Moffitt Cancer Center Physical Science-Oncology Center; the 4DB Center of Scripps Research Institute; and the Dana-Farber Cancer Institute Physical Science-Oncology Center.

The papers in this Special Topic can be categorized into one of three parts. In the first part, therapies directed toward CTCs in the bloodstream, and the responses of CTCs to cancer therapies, are considered. This subject is broadly reviewed by Greene et al. (2012) and then reviewed in the context of cancer stem cells by Faltas (2012). Li and King (2012) discuss cell adhesion receptors and how they can be targeted to neutralize CTCs. Liesveld (2012) discusses the targeting of myelogenous leukemia stem cells in the bloodstream, and Paolo Decuzzi and coworkers present data on the adhesion response of a model CTC cell line to curcumin treatment (Palange et al., 2012).

The second group of papers focuses on the basic science of CTCs, and how we might expect them to behave while in the circulation. Geng et al. (2012) show that the breast cancer biomarker MUC1 can act as a simultaneous adhesion counter-receptor for both endothelial E-selectin and ICAM-1, and they present a new model for the metastatic adhesion cascade paradigm. Burdick et al. (2012) consider not only the adhesion characteristics of CTCs to E-selectin, but propose that surface ligand expression may signal phenotypic changes of cancer stem cells. Rejniak (2012) presents a computational model that can be used to simulate the shear-induced deformation of CTCs in the bloodstream. In a pair of related papers, Tormoen et al. (2012b) pose the question of whether CTCs play a role in coagulation and thrombosis in the bloodstream, and then Lee et al. (2012) describe their theoretical model of procoagulant CTCs under flow.

The third and final part of this special issue is focused on the characterization of patient CTCs, including new methods dedicated to this goal. Another contribution from Tormoen et al. (2012a) presents new coagulation factor probes for the identification of procoagulant CTCs. Diamond et al. (2012) describe their characterization of prostate cancer CTCs isolated from patient blood. Garcia-Villa et al. (2012) measured the γ-H2AX levels in CTCs obtained from patients undergoing chemotherapy. Finally, a pair of papers from McCarty and coworkers describes the application of precise image analysis methods to measure the density and volume of patient CTCs in ovarian (Phillips et al., 2012b) and breast cancer (Phillips et al., 2012a), including a comparison with properties measured for normal blood cells.

The images featured on page 2 of this E-book, provided by Andrew Hughes of Cornell University, show viable CTCs isolated from a pancreatic cancer patient (upper image). The epithelial cancer cells stain positive for cytokeratin (red), and negative for the leukocyte marker CD45 (green), and show characteristically large nuclei stained in blue. The lower scanning electron micrograph shows a human leukocyte resting on a layer of halloysite nanotubes; these cells are unable to spread onto halloysite which enables efficient isolation of patient CTCs. This research is described in the lead paper by Greene et al. (2012).
